# Is the circadian clock a limit cycle oscillator?

**DOI:** 10.1186/1471-2202-12-S1-P220

**Published:** 2011-07-18

**Authors:** Jos HT Rohling, Johanna H Meijer

**Affiliations:** 1 Department of Molecular Cell Biology, Laboratory for Neurophysiology, Leiden University Medical Center, PO Box 9600, 2300 RC Leiden, The Netherlands

## 

Circadian rhythms are an essential property of living organisms, and arise from an internal clock. The circadian oscillator has many characteristics that are typical for a limit cycle oscillator, and limit cycle oscillator theory has often been used to model the circadian clock. In the past few years, it has become apparent that certain behaviors of the clock, such as adaptation to seasonal changes, are encoded by the complex interactions between the oscillatory neurons of the clock. The plasticity of the network is reflected in the amplitude of the rhythms of the electrical activity pattern of the clock observed in different photoperiods, as well as in its phase shifting capacity. From limit cycle theory, it is predicted that high-amplitude rhythms are more difficult to shift in phase than low-amplitude rhythms, in response to the same perturbation [1]. Yet, our investigation has shown that, surprisingly, oscillations with high amplitude have a large phase shifting capacity, and oscillations with low amplitude a small phase shifting capacity [2].

We performed a number of simulation studies where single cell oscillators could be perturbed according to a phase response curve (PRC). Short and long photoperiod waveform patterns were simulated by distributing 100 averaged single-unit electrical activity patterns in one cycle according to a narrow and a broad distribution. This resulted in population patterns resembling a narrow activity pattern with large amplitude for short winter days and a broad, low-amplitude pattern for long summer days. These distributions (see figure 1A) were used to distribute PRCs for the single units. Our study shows that distributions which coded for short photoperiod resulted in a high-amplitude population PRC, and distributions which coded for long photoperiod resulted in a low-amplitude population PRC (figure 1). These results indicate that at the population level, the attributes of the SCN clock cannot accurately be described by a limit cycle oscillator. These findings not only lead to new insights in the behavior of the biological clock, but are also relevant for the theory of oscillator networks. Importantly it is acknowledged that coupled limit cycle oscillators may reveal characteristics that are essentially different from the elements that make up the ensemble.

**Figure 1:**
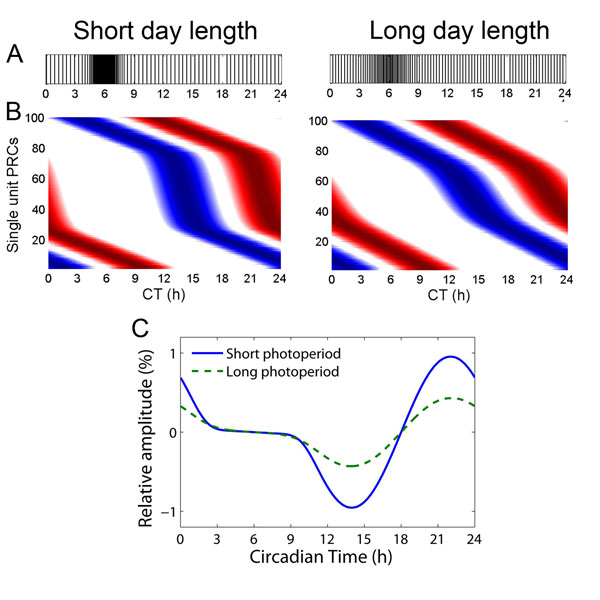
Short and long photoperiod PRCs obtained in simulations. (A) 100 neurons were distributed according to two Gaussian distributions; one distribution was based on observations in the SCN from short day length, and the other from long day length [1]. Each vertical line represents the peak time of a neuron. Many cells are active at CT 6, the middle of the day, and few cells are active at CT 18, the middle of the night. (B) The distributions in A were used to distribute 100 single unit PRCs. The y-axis represents differently phased single unit PRCs. The blue part of each line represents the delay part of the single unit PRC, the red part represents the advance part of the single unit PRC. The intensity of the color corresponds with the magnitude of the shift. The left figure shows the distribution for short days and the right figure shows the distribution for long days. (C) The resulting simulated population PRC for short and long days using single unit PRCs. The long day PRC shows lower amplitude than the short day PRC.

## References

[B1] Pittendrigh CS Kyner WT Takamura T The amplitude of circadian oscillations: temperature dependence, latitudinal clines, and the photoperiodic time measurement J Biol Rhythms 1993 6 299 313 1991 10.1177/074873049100600402 1773097

[B2] VanderLeest HT Houben T Michel S Deboer T Albus H Vansteensel MJ Block GD Meijer JH Seasonal encoding by the circadian pacemaker of the SCN Curr Biol 2007 17 468 473 10.1016/j.cub.2007.01.048 17320387

